# First successful radiofrequency ablation for papillary thyroid microcarcinoma in Indonesia: a case report

**DOI:** 10.1097/RC9.0000000000000159

**Published:** 2026-02-06

**Authors:** Kristanto Yuli Yarso, Muhana Fawwazy Ilyas, Mohamad Rifki Adli, Akmal Zhahir Wahyudi

**Affiliations:** a Department of Surgery, Oncology Division, Sebelas Maret University, Moewardi General Hospital, Surakarta, Indonesia; b Faculty of Medicine, Sebelas Maret University, Surakarta, Indonesia; c Department of Surgery, Sebelas Maret University, Moewardi General Hospital, Surakarta, Indonesia

**Keywords:** active surveillance, Indonesia, minimally invasive, papillary thyroid microcarcinoma, radiofrequency ablation, ultrasound-guided ablation

## Abstract

**Introduction:**

Papillary thyroid microcarcinoma (PTMC) is the most common subtype of thyroid cancer, generally associated with a favorable prognosis. Radiofrequency ablation (RFA) is emerging as a minimally invasive alternative to surgery, particularly for patients unsuitable for active surveillance and surgery. This report presents the first documented case of RFA for PTMC in Indonesia.

**Case report:**

A 40-year-old woman presented with a palpable thyroid nodule on the right side of her neck. Thyroid ultrasound and thyroid core needle biopsy confirmed PTMC. The patient underwent RFA using a 5-mm probe tip and 50-Watt power, employing the “moving shot” technique. The procedure was successful, with no reported complications. Follow-up assessments showed a significant reduction in tumor volume of 95.38%, from 0.264 ml to 0.012 ml, with no signs of progression at 12 months.

**Discussion:**

RFA provides a minimally invasive alternative to active surveillance with advantages, giving patient definitive treatment. Compare to surgery, this treatment have advantage such as shorter procedure time, lower procedural costs, reduced blood loss, and preservation of thyroid function, leading to faster recovery. However, wider adoption in Indonesia remains limited by the need for trained operators and access to advanced ultrasonography equipment. Strengthening workforce training, improving infrastructure, and generating long-term outcome data are essential for broader implementation.

**Conclusion:**

This case highlights RFA as a safe and effective treatment for PTMC, particularly for patients who do not wish to undergo active surveillance management for low risk thyroid cancer. Further studies are necessary to assess its long-term efficacy and feasibility.

## Introduction

Thyroid cancer is an endocrine malignancy that is becoming more common globally^[^1^]^. It is the most prevalent endocrine malignancy, with an incidence of 9/100 000 per year. Papillary thyroid carcinoma (PTC) is the most common form of thyroid cancer, comprising 70–90% of well-differentiated thyroid malignancies^[^2^]^. Papillary thyroid microcarcinoma (PTMC) is a subgroup of PTC, measuring 10 mm or less, which has also increased worldwide in recent years^[^3,4^]^.

Several studies have been conducted on the effect of radiofrequency ablation (RFA) on PTMC. A previous study showed that RFA could effectively eliminate low-risk PTMC with an exceedingly low incidence of complications^[^5^]^. Subsequently, ultrasound-guided RFA provides a benefit over active surveillance and surgery regarding quality of life. This supports the use of RFA as an alternate approach to active surveillance and surgery^[^6^]^. In addition, another study showed comparable 4-year clinical outcomes between RFA and thyroid lobectomy for low-risk PTMC^[^7^]^. Overall, RFA shows potential as a management option as a less invasive approach that could serve as an alternative to current treatment options for PTMC.

**HIGHLIGHTS**

- First documented RFA treatment for PTMC in Indonesia.
- Achieved 95.38% tumor volume reduction without complications.
- RFA demonstrated safe and effective alternative to surgery.
- Procedure used ultrasound-guided “moving shot” technique.
- Findings support wider implementation of RFA in low-resource settings.

However, to our knowledge, there have been no documented cases of RFA on PTMC from Indonesia. Furthermore, this case report illustrates the first successful application of RFA in a patient with PTMC in Indonesia. RFA for the thyroid has been performed since 2019 and has become the procedure of choice during the COVID-19 pandemic due to limited access to surgical services at that time.

## Case report

As this case involves patient materials and photographs, we report that it has been approved by our center’s ethical committee under approval number: 62/1/HREC/2025. Subsequently, the report has been prepared in accordance with the Surgical CAse REport (SCARE) 2025 guidelines^[^8^]^.

A 40-year-old woman presented to the Oncology Surgery Clinic at our center, having accidentally found a palpable lump on the right side of her neck since 2022. The patient noticed a small lump on the right side of her neck. She promptly sought medical evaluation at a local health facility within the same month of detection. The patient had no family history of thyroid disorders but there is history of breast malignancies and no significant psychosocial issues such as smoking or substance use. The patient had no history of chronic illnesses such as hypertension, diabetes mellitus, or autoimmune thyroid disorders, and no other systemic abnormalities were identified during preoperative evaluation.

In June 2022, following the initial detection of the lump in May 2022, the patient underwent an ultrasound for thyroid screening, which revealed a small lump in the right thyroid classified as TIRADS 4, indicating suspicion of malignancy. At the initial facility, the right thyroid nodule measured approximately 0.8 × 0.7 × 0.8 cm, which was comparable in size to the later assessment at our center. Two months later, an FNAB was performed under ultrasound guidance at a rural hospital, and the results showed a benign colloid nodule, classified as Bethesda System Category II. Given the discordant findings, the patient was referred to an oncology surgeon at our center. The patient opted for a non-surgical, minimally invasive procedure (RFA) after counseling on available management options.

On general examination, the patient was compos mentis and hemodynamically stable, with no signs of generalized pain. Local examination of the neck revealed a solitary, well-defined lump located on the right anterior aspect of the neck. According to the SOCRATES framework, the swelling had a gradual onset, first noticed 2 months before presentation. It was firm in consistency, non-tender, non-mobile, and moved with deglutition, without any radiation of pain. The patient reported no associated symptoms such as dysphagia, dyspnea, or hoarseness of voice, and there were no exacerbating or relieving factors identified. No additional masses were palpable on the left side of the neck, and no cervical lymphadenopathy was noted.

**Figure 1.**
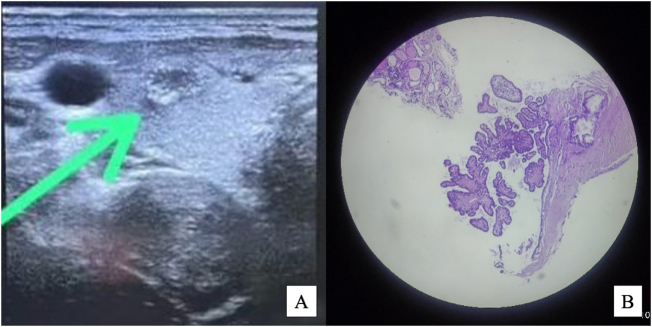
(A) Pre-ablation ultrasound showing a hypoechoic nodule in the right thyroid lobe with internal microcalcifications. (B) Preoperative core biopsy pathology confirming the diagnosis of papillary thyroid microcarcinoma (PTMC).

USG revealed a solid nodule with ill-defined margins, irregular contour, microcalcification, right thyroid lobe size 0.86 × 0.70 × 0.82 cm (0.26 cc) with vascularization, Ti-Rads 4, no suspicious lymphadenopathy (Fig. 1A). Pathology Thyroid FNAB: Benign Colloid Nodule (Bethesda System Category II). Pathology Thyroid core biopsy: thyroid biopsy at our center revealed thyroid tissue fragments with papillary follicular arrangement, lined with single-layer cuboidal epithelium, partially with a ground glass appearance, indicating PTMC (Fig. 1B).

**Figure 2.**
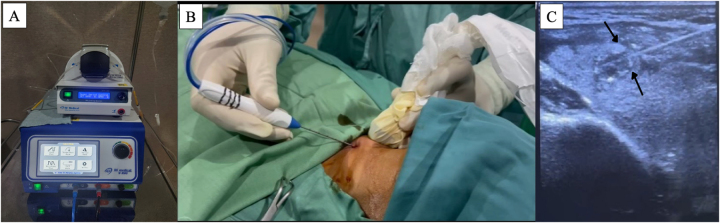
(A) Radiofrequency ablation (RFA) generator used during the procedure. (B) The needle for RFA (then inserted, with a trans isthmic approach, always displaying the needle along its major axis. (C) Ultrasound image during ablation showing a transient hyperechoic zone within the lesion (arrows), caused by tissue vaporization and microbubble formation, which confirms adequate thermal coverage of the tumor.

For the RFA procedure, the patient was placed under local anesthesia, and the operating field was sterilized and covered with a sterile drape. Lidocaine 2 cc was injected for local anesthesia. Cold 5% dextrose 10 cc was then injected to perform hydrodissection, separating the thyroid tissue from the strap muscles and carotid artery. A maximum power setting of 50W was used. A probe with a 5 mm tip was used to perform the ablation (Fig. 2A and B). The ablation technique followed the standard “moving shot” method, in which the nodule is ideally divided into smaller units that are treated independently. The electrode tip is first positioned in the deeper portion of the nodule, with gradual retraction toward more superficial areas. Since the tumor was small, ablation was completed in 30 seconds until power dropped and impedance increased. During ablation, a hyperechoic zone appeared within the tumor (Fig. 2C). The RFA procedure was performed by a surgical oncologist with prior hands-on training in thyroid ablative techniques and experience in ultrasound-guided interventions.

At the end of the procedure, before removing the needle, another examination was performed to assess the extension of the ablated area and compare it to the pre-treatment situation. The ablation proved complete, and the ablated area was clearly wider than the edge of the previously assessed neoplasm. The ablated area was limited to the thyroid gland only, with no ablation observed in surrounding structures such as the strap muscles, due to the effectiveness of the hydrodissection procedure.

**Figure 3.**
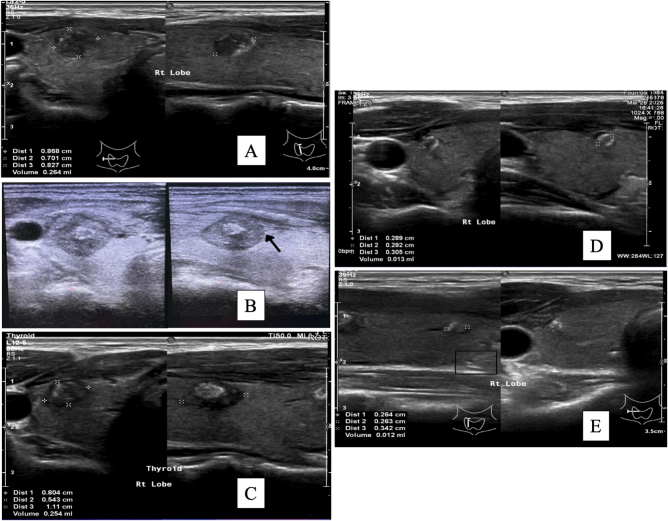
(A) Tumor appearance before ablation in sagittal and transverse views. (B) Tumor with ablated areas in transverse and sagittal views immediately after the procedure. (C) Six months post-ablation, showing necrotic areas around the tumor in transverse and sagittal views. (D) Nine months post-ablation, showing tumor shrinkage in transverse and sagittal views. (E) Twelve months post-ablation, near-complete tumor disappearance is observed in transverse and sagittal views.

The patient underwent follow-up evaluations at 6, 9, and 12 months post-procedure, for a total follow-up duration of 1 year. The results were favorable, with progressive tumor shrinkage observed over time (Fig. 3A–E). No complications such as hematoma or injury to surrounding organs were identified. The only reported side effect was mild pain lasting for three days, which was well managed with simple oral painkillers. Tumor volume reduction was noted at each follow-up interval, and the patient expressed satisfaction with the procedure.

**Figure 4.**
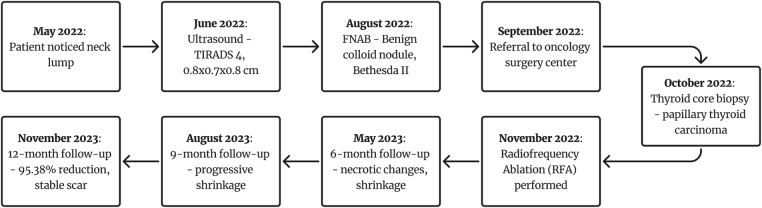
Timeline summarizing the patient’s clinical course from initial presentation to post-RFA follow-up evaluations.

Initially, the right thyroid lobe PTMC measured 0.86 × 0.70 × 0.82 cm (volume: 0.26 cc). At 6 months post-ablation, the tumor measured 0.80 × 0.54 × 1.11 cm (volume: 0.25 cc), indicating early shrinkage. At 12 months post-ablation, the tumor had further shrunk to 0.26 × 0.26 × 0.34 cm (volume: 0.01 cc), representing a volume reduction of approximately 95.38%. A summary of the chronological course, including presentation, diagnosis, treatment, and follow-up, is illustrated in Figure 4.

The patient expressed satisfaction with the overall treatment experience, highlighting the quick recovery and the absence of surgical scars. She reported relief that the procedure could be performed under local anesthesia and appreciated being able to resume daily activities within a few days. Overall, she felt reassured by the follow-up results and expressed confidence in the outcome of RFA.

## Discussion

RFA has emerged as an effective treatment for PTMC, and its success has been demonstrated in various studies and individual cases. A previous study showed that in the 142 patients with solitary T1N0M0 PTC, the tumor disappearance rate after RFA was reported at 53.5%, with a disease progression rate of 2.1% and complications occurring in only 2.8% of cases^[^9^]^. Subsequently, another study focusing on bilateral PTMC found a complete disappearance rate of 92% after a mean follow-up of approximately 48 months, with a volume reduction ratio (VRR) of 99.94%^[^10^]^. Regarding volume reduction, the mean volume of tumors treated by RFA decreased significantly from an initial average of 75.22 mm^3^ to just 0.09 mm^3^ post-treatment^[^10^]^. In addition, separate analysis indicated that among 152 tumors treated, with the mean volume reduction was nearly 100% at 3-year post-RFA^[^11^]^.

The follow-up duration varied across studies, with one reporting an average follow-up of 39 months, during which no local recurrences were observed, and there was only a 3% complication rate overall^[^11^]^. In another cohort, the median follow-up was around 7.8 months, showing significant reductions in tumor volumes at multiple intervals post-ablation (96.20% reduction at 12 months) for PTMC patients^[^12^]^. Subsequently, regarding safety profiles, the overall complication rate associated with RFA procedures was low, with serious complications being rare. For instance, one study reported only a 3% complication rate, including minor issues such as voice changes^[^11^]^. Additionally, in the study involving bilateral PTMC, no life-threatening complications or delayed adverse events were recorded during the follow-up period^[^10^]^. The incidence of lymph node metastasis post-RFA was also noted to be low, at around 2.13%, with only two cases of recurrence reported among patients undergoing long-term monitoring^[^10^]^.

This study reported a case that represents the first documented successful application of RFA for PTMC in Indonesia, contributing valuable insights into its viability as a treatment option. The patient, diagnosed with solitary right-lobe thyroid nodules demonstrating moderately suspicious characteristics (TIRADS 4) and prominent vascularization, presented a complex clinical profile involving suspected thyrotoxicosis and no lymphadenopathy. Pre-procedural assessments highlighted nodules with microcalcifications and irregular contours, aligning with characteristics commonly associated with PTMC. The bilateral nature and intra-nodular vascularization raised considerations for a less invasive approach to treatment. RFA was selected based on global studies indicating RFA’s efficacy and safety as a treatment for low-risk PTMC, comparable to traditional thyroid lobectomy but with improved quality of life outcomes^[^5,6^]^.

According to the 2025 American Thyroid Association (ATA) guidelines, percutaneous thermal ablation, using radiofrequency (RFA), microwave (MWA), or laser ablation (LA), has emerged as a potential primary treatment option for low-risk PTC in carefully selected patients. The ATA notes that selection criteria are similar to those used for active surveillance and that such minimally invasive approaches may be appropriate for patients who decline or are unsuitable for surgery but are uncomfortable with surveillance^[^13^]^. Similarly, the European Society for Medical Oncology (ESMO) guidelines support a risk-adapted approach, recommending active surveillance for unifocal intrathyroidal PTMC ≤10 mm without nodal or extrathyroidal involvement, reserving surgery for higher-risk or multifocal disease^[^14^]^. In line with these recommendations, the present patient opted for a non-surgical, minimally invasive procedure (RFA) after being counseled on available management options. In this context, RFA can be positioned within the spectrum of accepted, minimally invasive management strategies for low-risk PTMC, serving as an alternative for patients who are ineligible for or unwilling to undergo surgical resection and uncomfortable with active surveillance procedure.

Beyond RFA, PTMC can be managed with several established approaches, including active surveillance, thyroid lobectomy, total thyroidectomy, and radioactive iodine (RAI) therapy. International guidelines (e.g., the 2015 American Thyroid Association) emphasize risk stratification when selecting therapy^[^15^]^. Table 1 summarizes the key management modalities for PTMC, their typical indications, outcomes, and the advantages/limitations of each approach.

**Table 1:** Management options for papillary thyroid microcarcinoma (PTMC)

Treatment modality	Indications	Efficacy & outcomes	Advantages	Limitations
Active surveillance (AS)	Low-risk PTMC ≤ 1 cm, intrathyroidal, no LN spread/aggressive features, and often older/comorbid patients or those avoiding surgery^[^15^]^	3–5% tumor growth, 1% new LN metastasis (5–10 years), 99–100% survival, high quality of life^[^16^]^	Avoids overtreatment and surgical risk^[^17^]^, preserves thyroid function, and remain reversible if progression occurs^[^18^]^	Requires lifelong monitoring, may cause anxiety, and 5–15% may later need surgery^[^18^]^
Thyroid lobectomy	Unifocal low-risk PTMC ≤ 1 cm confined to one lobe, without extrathyroidal or distant spread^[^15^]^	99% survival, 6–8% recurrence (20–40 years), and excellent quality of life^[^15,18^]^	Less invasive with short recovery, lower risk of hypoparathyroidism, and thyroid function often preserved^[^19^]^	Small recurrence risk in remaining lobe, RAI not feasible without completion surgery, and standard surgical risks remain^[^20^]^
Total thyroidectomy	High-risk or multifocal disease (bilateral tumors, extrathyroidal extension, LN metastasis, aggressive histology)^[^15^]^ and patients requiring RAI^[^21^]^	98–99% disease-free survival, 0.3–0.5% mortality, and good long-term quality of life with hormone therapy^[^15^]^	Provide definitive tumor removal, enable RAI therapy, simplify follow-up, and treat multifocal/bilateral disease^[^22^]^	Causes permanent hypothyroidism, risk of hypoparathyroidism and nerve injury, longer recovery, and possible overtreatment in low-risk PTMC^[^18^]^
Radiofrequency ablation (RFA)	Low-risk PTMC ≤ 1 cm confined to the thyroid, suitable for non-surgical or surgery-averse patients^[^23^]^	Meta-analysis (1.770 patients) showed 79% complete ablation, 1.5% progression, 0.4% recurrence^[^23^]^, and outcomes comparable to surgery	Minimally invasive, outpatient, scar-free, preserves thyroid function, and has a very low complication rate^[^23^]^	Limited long-term data, requires skilled operator/equipment, and not indicated for high-risk disease^[^23^]^
Radioactive iodine (RAI) therapy	Adjuvant after total thyroidectomy for high-risk or select intermediate-risk disease; not indicated for low-risk PTMC^[^21^]^	Improves recurrence-free survival in high-risk cases but offers no benefit for low-risk PTMC^[^24^]^	Ablates microscopic residual disease, improves follow-up accuracy, and is a safe, well-established adjunct therapy^[^21^]^	Requires prior total thyroidectomy, may cause radiation side effects (dry mouth, taste changes), lifestyle isolation post-therapy, and unnecessary in low-risk disease^[^24^]^

Compared with conventional management options such as active surveillance, lobectomy, total thyroidectomy, and RAI therapy, RFA offers a minimally invasive alternative with comparable oncologic control and superior safety. The advantages of RFA include its minimally invasive nature, repeatability, and low morbidity, making it suitable for carefully selected patients who decline active surveillance and surgery^[^13^]^. Clinical data support this position, with previous study reporting that RFA achieved a mean tumor volume reduction of 98% without recurrence or major complications in low-risk PTMC patients ineligible for surgery^[^25^]^. Likewise, long-term follow-up findings have confirmed durable outcomes, demonstrating 100% local tumor disappearance at 5 years with no evidence of recurrence, nodal spread, or delayed surgery^[^26^]^. Compared with surgical approaches, RFA provides comparable disease control with fewer complications, faster recovery, improved quality of life, and lower cost, positioning it as a safe, effective option for selected low-risk PTMC cases.

Post-RFA imaging demonstrated a significant reduction in nodule size, with the right lobe nodule volume decreasing from 0.26 cc to approximately 0.012 cc. These results align with the findings of the existing literature that support RFA’s capacity for targeted ablation, minimal invasiveness, and preservation of thyroid function^[^7^]^. The absence of post-procedural complications further underscores RFA’s safety profile in the treatment of PTMC. Although residual nodules remained post-ablation, their reduced size and the lack of new suspicious lymphadenopathy indicate effective disease control. These findings are especially significant in regions with limited access to advanced surgical treatments, as RFA can be performed under ultrasound guidance with minimal resources.

The 95.38% reduction in tumor volume indicates effective ablation, consistent with previously reported outcomes showing continued tumor shrinkage for several months after RFA. No regrowth or new nodules were detected during serial imaging, suggesting stable post-ablation changes. Since RFA has already been adopted in the treatment protocol for low-risk PTMC, there is no concern in continuing observation until the tumor completely disappears, as the shrinkage effect may persist for up to 2 years after the procedure. Moreover, follow-up evaluation showed no enlargement of the residual area, indicating that it represents post-RFA scar tissue rather than viable tumor. Follow-up will continue for at least 2 years with periodic neck ultrasound to monitor for recurrence or residual disease, as nodule shrinkage and scar formation may still progress during this period.

Although the 12-month follow-up demonstrated a promising local response, longer surveillance is essential to validate the oncologic safety and durability of RFA in PTMC. Multi-year follow-up is necessary to confirm sustained tumor control and to identify potential late recurrences.

This case report is limited by its single-patient design involving a low-risk, unifocal PTMC and a relatively short 1-year follow-up, which may not capture late recurrences or long-term outcomes. Therefore, the findings may not be generalizable to patients with multifocal, bilateral, or high-risk disease. RFA is best suited for low-risk, intrathyroidal lesions without lymph node metastasis, extrathyroidal extension, or aggressive histological variants. Patients with multifocal or extensive disease are more appropriately managed with surgical resection. Further long-term studies with larger patient cohorts are needed to confirm the oncologic safety and durability of RFA outcomes.

## Conclusion

This case illustrates the successful implementation of ultrasound-guided RFA for PTMC in Indonesia, marking a milestone in local endocrine oncology management. The significant reduction in nodule size, coupled with the absence of suspicious lymphadenopathy or procedural complications, supports RFA as a viable, minimally invasive treatment alternative to active surveillance and surgery. The positive outcome in this patient advocates for the broader adoption of RFA in clinical practice for low-risk PTMC. Further research and follow-up studies are necessary to solidify these findings and optimize RFA protocols for broader patient populations, especially in Indonesia.

## Data Availability

Data supporting the findings of this study are available from the corresponding author upon reasonable request, subject to ethical and privacy considerations.
